# miR-25-3p protects renal tubular epithelial cells from apoptosis induced by renal IRI by targeting DKK3

**DOI:** 10.1515/biol-2021-0127

**Published:** 2021-12-31

**Authors:** Yu Zhang, Xiangrong Zuo

**Affiliations:** Department of Critical Care Medicine, The First Affiliated Hospital of Nanjing Medical University, Nanjing, Jiangsu, People’s Republic of China; Department of Critical Care Medicine, The First Affiliated Hospital of Nanjing Medical University, No.300 GuangZhou Road, Nanjing, Jiangsu, People’s Republic of China

**Keywords:** miR-25-3p, renal ischemia-reperfusion injury, apoptosis, DKK3

## Abstract

Renal ischemia-reperfusion injury (IRI) is one of the main causes of acute kidney injury (AKI). So far, there have been many studies on renal IRI, although an effective treatment method has not been developed. In recent years, growing evidence has shown that small noncoding RNAs play an important regulatory role in renal IRI. This article aims to explore whether microRNA-25-3p (miR-25-3p) plays a role in the molecular mechanism of renal IRI. The results showed that the expression level of miR-25-3p was significantly downregulated in a rat renal IRI model, and this result was confirmed with in vitro experiments. After the hypoxia-reoxygenation treatment, the apoptosis level of NRK-52E cells transfected with miR-25-3p mimics decreased significantly, and this antiapoptotic effect was antagonized by miR-25-3p inhibitors. In addition, we confirmed that DKK3 is a target of miR-25-3p. miR-25-3p exerts its protective effect against apoptosis on NRK-52E cells by inhibiting the expression of DKK3, and downregulating the expression level of miR-25-3p could disrupt this protective effect. In addition, we reconfirmed the role of miR-25-3p in rats. Therefore, we confirmed that miR-25-3p may target DKK3 to reduce renal cell damage caused by hypoxia and that miR-25-3p may be a new potential treatment for renal IRI.

## 1 Introduction

Ischemia-reperfusion injury (IRI) is an inevitable injury during kidney transplantation. At present, renal IRI is one of the main causes of acute kidney injury (AKI) and the most important factor of delayed graft function [1]. The frequency of ARF among patients is 2–5% during hospitalization, and acute tubular necrosis (ATN) is the main cause of ARF, accounting for 38% and 76% of cases of ARF in inpatients and ICU patients, respectively [2]. In addition, patients may still develop long-term chronic kidney disease [3]. In recent years, research on renal IRI has gradually increased, and its pathogenesis has gradually been revealed. It is currently believed that the pathogenesis of renal IRI mainly involves intracellular calcium overload, large oxygen-free radical accumulations, and microcirculation disorders [4,5]. In recent years, studies have shown that the loss of renal tubular epithelial cell function induced by apoptosis is crucial in the development of renal IRI. However, renal IRI still lacks effective treatments to prevent or reduce kidney damage.

MicroRNA (miRNA) is a short single-stranded non-coding RNA molecule with a length ranging from 21 to 25 nucleotides. miRNAs can simultaneously bind to the mRNAs of multiple target genes and inhibit posttranscriptional translation, thus playing an important regulatory role in the body [6]. In recent years, an increasing number of studies have shown that miRNAs, as key regulators, play an important role in regulating the cell cycle, proliferation, differentiation, growth, and apoptosis. Moreover, in the field of nephropathy, a large number of studies have shown that miRNAs play an important role in renal fibrosis, renal cell carcinoma, and diabetic nephropathy and have the potential for use in new treatments. For example, miR-21, miR-29, and miR-192, as key regulators, play an important regulatory role in the progression of renal fibrosis [7–9]. Related studies have shown that many miRNAs (such as miR-15a and miR-210) may become new tools for the detection, treatment, and prognosis of renal cancer [10–12]. Moreover, miR-34a-5p, miR-27a, and miR-126 have also been shown to be involved in the development of diabetic nephropathy [13–16]. For renal IRI, miRNA is also a hot spot that many scholars have paid attention to in recent years. miR-25-3p has been proven to exert cardioprotective effects by targeting high-mobility group box 1 in myocardial ischemia-reperfusion injury [17]. In addition, we found that the expression level of miR-25-3p was significantly downregulated in the rat renal IRI model in previous studies, and this result was also confirmed via *in vitro* experiments. However, research has not revealed the role that miR-25-3p plays in renal IRI. Therefore, this study aimed to determine the role of miR-25-3p in renal IRI and explore its molecular mechanism.

## 2 Materials and methods

### 2.1 Animal

Sprague-Dawley rats (4–5 weeks of age) weighing 180–220*g* were purchased from the Centre of Experimental Animals at Wuhan University Medicine College (Hubei, China). All rats were kept in a standard temperature-controlled room, and a 12 h light-dark cycle was implemented. All rats were provided food and water ad libitum and allowed to adapt to the environment for 1 week. All rats were randomly divided into seven groups as follows: sham operation group, which only received the sham operation treatment; 30 min ischemia/0 h reperfusion group, which only received renal pedicle clamping for 30 min; 30 min ischemia/12 h reperfusion group, which received renal pedicle clamping for 30 min and then restored renal blood flow for 12 h; 30 min ischemia/24 h reperfusion group (IRI group), which received renal pedicle clamping for 30 min and then restored renal blood flow for 24 h; 30 min ischemia/48 h reperfusion group, which received renal pedicle clamping for 30 min and then restored renal blood flow for 48 h; I/R + None group, which was injected with normal saline; and I/R + ad-miR-25-3p NC group, which was transfected with adenovirus carrying control scrambled short hairpin RNA (miR-Con) 3 days before the renal ischemia-reperfusion treatment. The I/R + ad-miR-25-3p group was transfected with adenovirus carrying miR-25-3p (1 × 10^9^ plaque-forming units) by tail vein injection 3 days before renal ischemia-reperfusion treatment (*n* = 6 in each group).


**Ethical approval:** The research related to animal use has been complied with all the relevant national regulations and institutional policies for the care and use of animals and was approved by the ethics committee of the Nanjing Medical University.

### 2.2 Renal IRI model

The rats were fasted overnight and anesthetized with an intraperitoneal injection of 3% sodium pentobarbital (0.1 mL/100 g body weight), and a midline abdominal incision was made. An electric heating pad was used to keep the rat’s body temperature constant, and care was taken to prevent the rat from being burned. The renal pedicle was dissected, and a nontraumatic clamp was used to clamp the renal pedicle for 30 min. Next, the renal pedicle was dissected, and a nontraumatic clamp was used to clamp the renal pedicle for 30 min [18]. Then, the clamp was removed, and the blood supply to the kidneys was restored (0, 12, 24, 48 h). In the sham control group, the rats only received an abdominal incision without clamping the renal pedicle. Finally, all rats were euthanized by decapitation, and their kidney tissues were carefully dissected for subsequent experiments. Each group contains six rats.

### 2.3 Cell culture and hypoxia-reoxygenation (H/R) treatment

Renal tubular epithelial NRK-52E cells (the number of passages of the cells is between 5 and 10) were cultured in high-glucose Dulbecco’s modified Eagle’s medium (DMEM) supplemented with 10% fetal bovine serum penicillin (100 U/mL) and streptomycin (100 U/mL). To simulate a hypoxic environment, the cells were placed in a tri-gas incubator containing 94% N2, 5% CO_2_, and 1% O_2_ for 24 h and then transferred to a normal environment (5% CO_2_, 21% O_2_, and 74% N_2_). The cells are then collected for RNA isolation, protein extraction, and many other experiments.

### 2.4 Cell transfection

The miR-25-3p mimic, miR-25-3p inhibitor, and corresponding negative controls were purchased from RiboBio (Ribo, China). When the degree of cell aggregation reached 60–70%, the mimic, inhibitor, and corresponding negative controls were transfected into NRK-52E cells using Lipofectamine 3000 (Life Technologies, USA) according to the manufacturer’s protocol.

### 2.5 Recombinant adenoviruses

Recombinant adenoviruses for the expression of miR-25-3p or control scrambled short hairpin RNA were generated using the BLOCK-iT adenoviral RNAi expression system (Invitrogen; Thermo Fisher Scientific, Inc.) according to the manufacturer’s protocol. The virus was purified using an Adeno-XTM Virus Purification Kit (BD Biosciences; Clontech, Mountain View, CA) and then diluted in PBS. The virus solution was injected into mice via the tail vein at 2 × 10^12 ^g/mL. After 3 weeks of observation, the expression level of miR-25-3p was upregulated by about two times by qPCR. After the transfection became stable, a renal ischemia-reperfusion injury model was constructed.

### 2.6 Haematoxylin–eosin staining

Renal tissue was fixed in 4% phosphate-buffered formalin (pH 7.4) for 24 h, dehydrated, and then embedded in paraffin according to conventional protocols. Then, 3 µm-thick paraffin sections were stained with hematoxylin (0.2%) and eosin (1%). To ensure the objectivity of the observation results, all slices were evaluated by experienced pathologists who were blinded to the experimental groupings.

### 2.7 Measurement of MDA and SOD in kidney tissue

Tissue samples were first homogenized with an Ultra-Turrax system (T25, IKAH-Labourtechnik, Staufen, Germany) in Tris buffer (pH 7.4) and then centrifuged at 13,000*g* (Heraeus Biofuge Primo R, Karlsruhe, Germany) at 4°C for 20 min, after which the supernatants were collected. The xanthine oxidase method and TBA (thiobarbituric acid) method were used to detect the SOD and MDA levels, and the corresponding SOD and MDA assay kits were purchased from Nanjing Jiancheng Bioengineering Institute (Jiangsu, China). The absorbance was detected at 450 or 532 nm using an automatic microplate reader (Multiskan MK3, Thermo Scientific). The SOD levels were expressed as U/mg protein in tissue, and the MDA levels were expressed as nmol/mg protein in the tissue.

### 2.8 Cell Counting Kit-8 (CCK-8)

NRK-52E cells were cultured in groups according to the experimental plan, and Cell Counting Kit-8 (Dojindo, Beijing, China) was used to detect all cells in the logarithmic growth phase and calculate cell viability. The cell proliferation rate was calculated as follows:

\begin{array}{l}(\text{Absorbance}\hspace{.25em}\text{value}\hspace{.25em}\text{at}\hspace{.25em}450\hspace{.25em}\text{nm}\hspace{.25em}\text{of}\hspace{.25em}\text{the}\hspace{.25em}\text{experimental}\hspace{.25em}\text{group}\\ \hspace{1em}\mbox{--}\hspace{.25em}\text{Absorbance value}\hspace{.25em}\text{at}\hspace{.25em}450\hspace{.25em}\text{nm}\hspace{.25em}\text{of}\hspace{.25em}\text{the}\hspace{.25em}\text{control}\hspace{.25em}\text{group})\\ \hspace{1em}/\text{Absorbance}\hspace{.25em}\text{value}\hspace{.25em}\text{at}\hspace{.25em}450\hspace{.25em}\text{nm}\hspace{.25em}\text{of}\hspace{.25em}\text{control}\hspace{.25em}\text{group}\times 100 \% \end{array}]

### 2.9 Flow cytometry analysis

To detect the apoptosis rate, an Annexin V-fluorescein-5-isothiocyanate (Annexin V-FITC) apoptosis detection kit (Sigma. St Louis, MO, USA) was used to analyze phosphatidylserine exposure according to the manufacturer’s protocol. After reaching 80% confluence, the cells from different groups were collected and digested with trypsin without EDTA, washed twice with cold PBS, and resuspended in buffer. Then, the cells were resuspended in 500 µL 1 × binding buffer and incubated with 5 µL Annexin and 5 µL propidium iodide (PI) in the dark at room temperature for approximately 15 min. Finally, flow cytometry (BD FACS Calibur, USA) was performed to detect and quantify the apoptotic cells.

### 2.10 Western blot analysis

Protein samples were extracted from cultured cells and kidney tissue with protein lysis RIPA buffer (Solarbio, Beijing, China) supplemented with the protease inhibitor phenylmethylsulfonyl fluoride (PMSF) and phosphatase inhibitor (Solarbio, Beijing, China) according to the manufacturer’s protocol. After 30 min of lysis on ice, lysates were collected by centrifugation at 12,000 rpm for 20 min at 4°C. Protein concentrations were determined using a bicinchoninic acid (BCA) assay kit (Beyotime Institute of Biotechnology, Haimen, China). Protein samples in each group (40 µg per lane) were subjected to 10 or 15% sodium dodecyl sulfate-polyacrylamide gel electrophoresis (SDS-PAGE) and then transferred to a polyvinylidene fluoride membrane (PVDF) for 1–2 h at 200 mA according to the molecular weight. The membrane was blocked with TBST containing 5% dried skim milk for 1 h at room temperature and then immunoblotted with primary antibodies at 4°C overnight. The primary antibodies used were as follows: Bax (1:2,000, Abcam, ab182733); Bcl-2 (1:2,000, Proteintech, 26593-1-AP); and cleaved caspase-3 (1:1,000, Cell Signaling Technology, 9661). Then, the membrane was washed with TBST and incubated with anti-mouse/rabbit secondary antibody (P/N 925-32210, P/N 925-32211, 1:10,000, LI-COR Biosciences) conjugated to IRDye 800CW for approximately 1 h at room temperature. The signal was finally quantified by a western blot detection system (Odyssey Infrared Imaging, LI-COR Biosciences).

### 2.11 Quantitative reverse-transcription polymerase chain reaction (qRT-PCR)

Total RNA was extracted from cultured cells and kidney tissue using the mirVana kit (Ambion, Austin, TX) and then reverse-transcribed into cDNA using a Takara RNA PCR kit (Takara Biotechnology, Dalian, China). Real-time PCR was performed with the 7900 HT Real-Time PCR System (Applied Biosystems Life Technologies, Foster City, CA, USA) for 40 cycles with GAPDH as an internal control. The TaqMan miRNA assay kit (Applied Biosystems) was used to detect the relative expression of miR-25-3p. The small nuclear RNA U6 was used as an internal control to normalize the expression of miR-25-3p. Finally, the 2^−ΔΔCt^ values were used to calculate the fold change.

### 2.12 Luciferase reporter assay

The DKK3 3′UTR carrying a miR-25-3p binding site was constructed by PCR and subsequently cloned into the pMIR-REPORT vector to construct the wild-type DKK3 (DKK3-WT) luciferase reporter construct. Then, the DKK3-WT construct and miR-25-3p mimic or scrambled sequence oligonucleotides were co-transfected into NRK-52E cells using Lipofectamine 3000 (Life Technologies, USA). After 48 h of transfection, luciferase activity was analyzed using a Dual-Luciferase Reporter Assay System (Promega, Madison, WI, USA). All procedures were performed according to the manufacturer’s instructions.

### 2.13 Immunohistochemical (IHC) staining

Kidney tissues were fixed in 10% neutral-buffered formalin solution for 48 h and transferred to 70% ethanol. Tissues were processed and embedded in paraffin. Samples were cut into 5 µm-thick sections, deparaffinized in xylene, and rehydrated using a decreasing ethanol gradient followed by 0.1 M PBS. The tissues were then sequentially blocked with 0.3% hydrogen peroxide in methanol for 15 min and with 5% goat serum in PBS for 30 min at room temperature. The tissue sections were first incubated with the primary antibodies Bax (1:250, Abcam, ab32503) or Bcl-2 (1:70, Abcam, ab194583) in 1% goat serum at 4°C overnight. The slides were then sequentially incubated with a biotinylated anti-rabbit secondary antibody (Vector Laboratories) for 1 h and with horseradish peroxidase streptavidin (Vector Laboratories, SA-5004) for 30 min at room temperature before being visualized with a DAB kit (Vector Laboratories, SK-4100). Finally, sections were counterstained with hematoxylin and observed by two independent pathologists. The percentage of stained target cells was evaluated in 10 random microscopic fields per tissue section, and their averages were subsequently calculated.

### 2.14 TUNEL staining

Apoptosis scores of rat kidney tissue in different groups were determined with terminal transferase-medicated dUTP nick-end labeling (TUNEL) staining using an *In Situ* Apoptosis Detection kit (Roche Applied Science, Basel, Switzerland). Apoptotic nuclei were stained brown, and negative nuclei were stained blue. The TUNEL assay was performed according to the manufacturer’s instructions. Cell counting was performed under a light microscope, and the degree of cell apoptosis was expressed as the percentage of positive cells.

### 2.15 Statistical analysis

The experimental data are presented as the mean ± standard deviation (SD). The significance of differences between data was evaluated using one-way analysis of variance (ANOVA) with SPSS 19.0 statistical software (SPSS Inc., Chicago, IL), and *P* < 0.05 was considered significant. All graphs shown in this manuscript were constructed with GraphPad Prism 5.0 software. The experimental data were obtained from three experimental replicates.

## 3 Results

### 3.1 Renal IRI induces apoptosis

**Figure 1:**
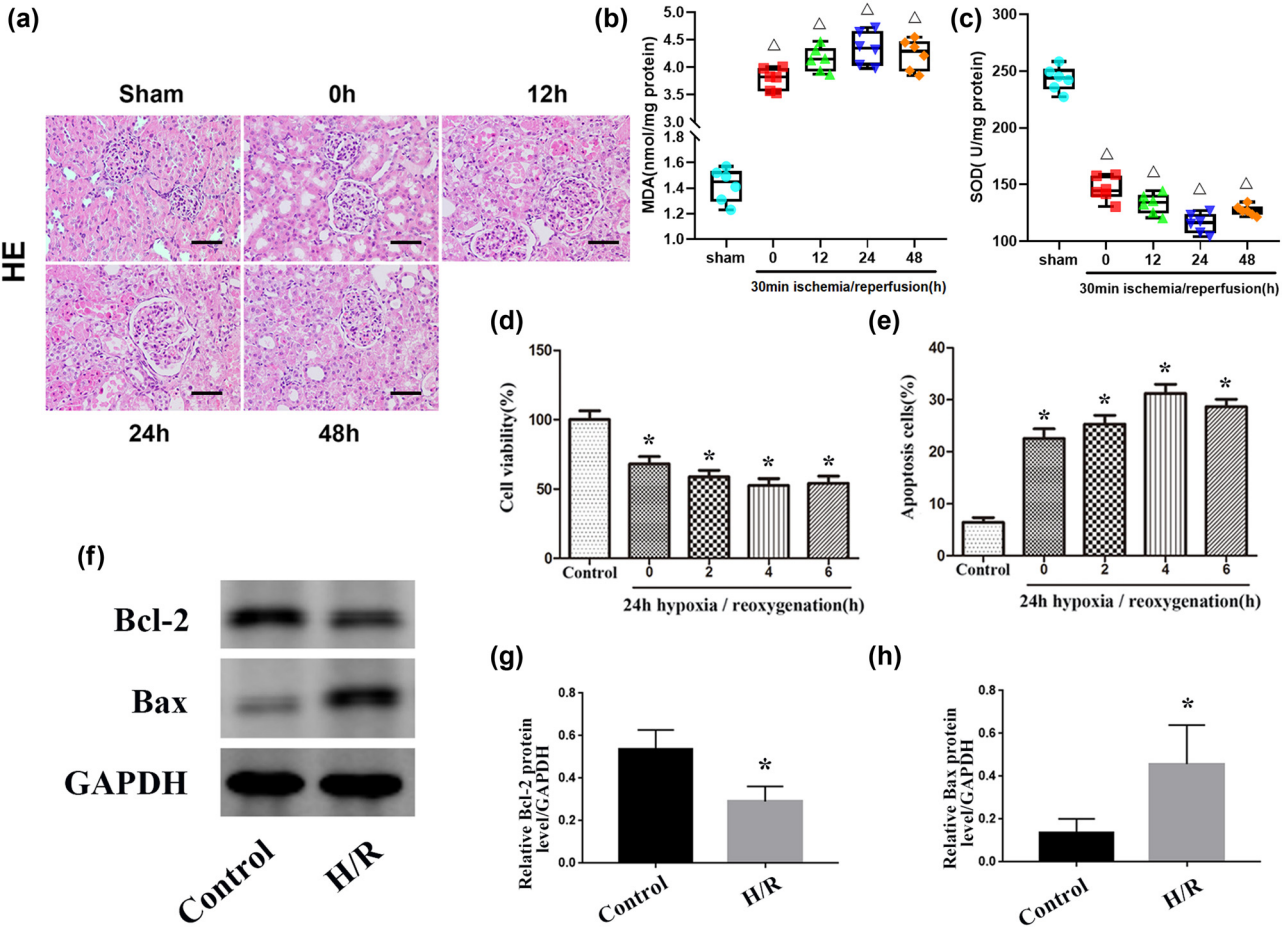
IRI induces renal apoptosis. (a) HE staining of kidney tissues from the sham and IRI groups. Scale bar, 50 µm. (b and c): Levels of MDA and SOD in kidney tissue in the sham and IRI groups. (d) Quantitative analysis of cell proliferation levels. (e) Quantitative analysis of the percentage of apoptotic cells by flow cytometry. (f) Expression levels of apoptosis-related proteins (Bax and Bcl-2) in the control and H/R groups. (g and h) Quantitative values of the relative expression levels of the Bax and Bcl-2 proteins in the control and H/R groups. The data are expressed as the mean ± SD. Δ*P* < 0.05 versus the sham group, *P < 0.05 versus the control group.

To study the cytopathological changes during renal IRI, Sprague-Dawley rats were selected to establish renal IRI models, and they were divided into an experimental group (IRI group) and a sham operation group (sham group). In the IRI group, after the renal pedicle was clamped for 30 min, the rats were divided into a 0 h group, 12 h group, 24 h group, and 48 h group according to the subsequent recovery time of renal perfusion. In the control group, the rats only underwent the sham operation. The results of HE staining showed that obvious histopathological changes did not occur in the sham group while pathological damage of rats in the renal IRI group manifested mainly as renal tubular cell swelling, extensive tubule dilatation, and degeneration, and the pathological damage was the most serious after renal perfusion was restored for 24 h (Figure 1a). To assess the level of oxidative stress in kidney tissue, we measured the SOD and MDA levels (Figure 1b and c). The results showed that MDA levels were increased and SOD levels were decreased after the renal pedicle was clamped for 30 min, and this change was most obvious after renal perfusion was restored for 24 h. Moreover, NRK-52E cells were cultured *in vitro* in an anoxic environment for 24 h to simulate ischemia within the tissue and then divided into 0 h, 2 h, 4 h, and 6 h groups according to the time of resuming oxygen supply. Subsequently, CCK-8 experiments and flow cytometry analyses were performed, and the results showed that cell proliferation was inhibited, and apoptosis was significantly increased after culture in an anoxic environment, and this change was most obvious after 4 h of reoxygenation (Figure 1d and e). Western blot analysis showed that the protein expression levels of Bax in the H/R group were significantly higher compared with the control group, while the expression levels of Bcl-2 were significantly decreased in the H/R group (Figure 1f–h). Together, these results indicate that with the increase in oxidative stress levels, cell growth was inhibited, and the degrees of renal injury and cell apoptosis were significantly increased during renal IRI.

### 3.2 Hypoxia inhibits miR-25-3p expression in NRK-52E cells

**Figure 2:**
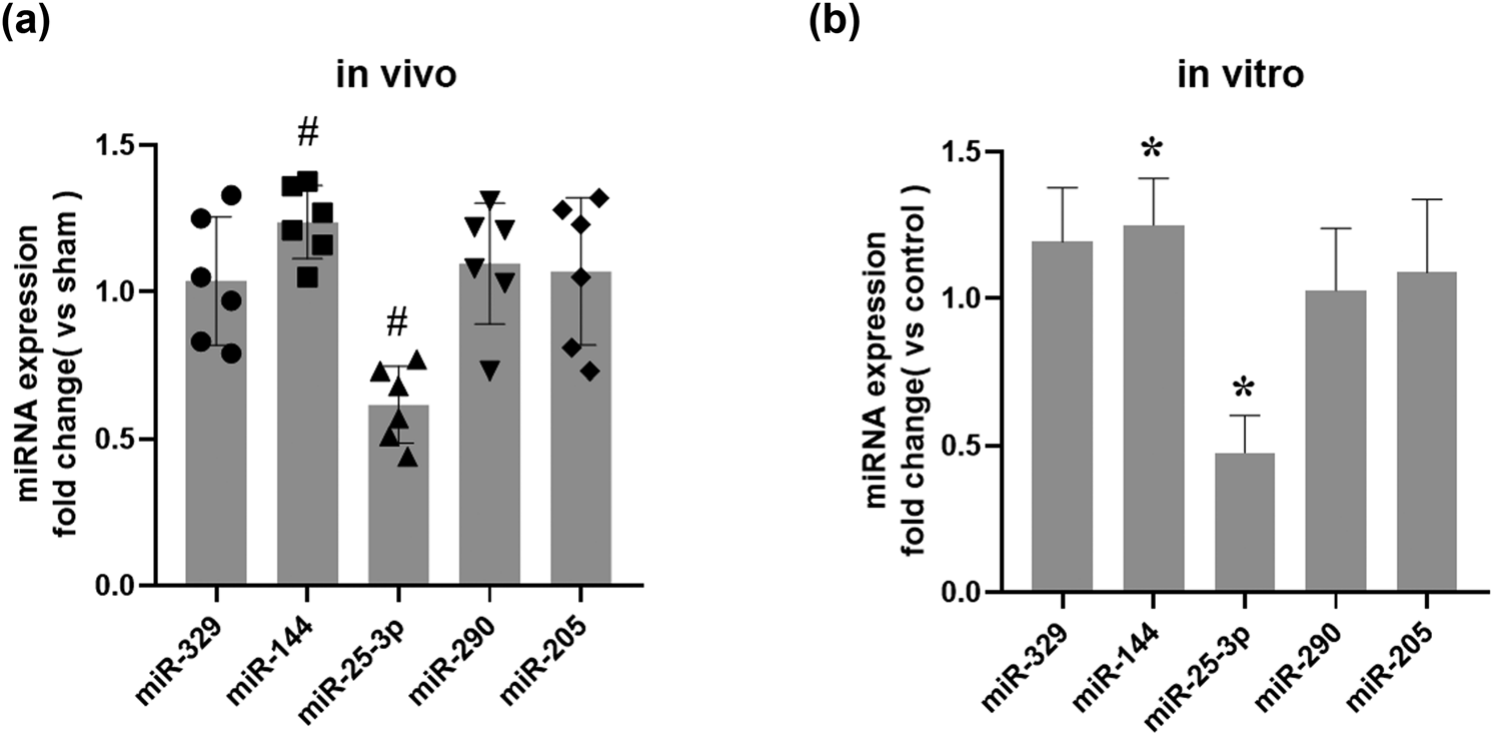
The expression level of miR-25-3p is significantly down-regulated in renal IRI and NRK-52E cells that have undergone H/R treatment. (a) Fold changes in the expression levels of miR-329, miR-144, miR-25-3p, miR-290, and miR-205 in the IRI group compared to the sham group. (b) Fold changes in the expression levels of miR-329, miR-144, miR-25-3p, miR-290, and miR-205 in the H/R group compared to the control group. The data are expressed as the mean ± SD. ^#^ *P* < 0.05 versus the sham group, *P < 0.05 versus the control group.

To explore the role of microRNAs in renal IRI, we screened several microRNAs that may be related to the renal IRI process, including miR-329, miR-144, miR-25-3p, miR-290, and miR-205, and performed qRT-PCR assays to detect the levels of these five microRNAs in both normal and IRI kidney tissues. The results showed that the expression level of miR-144 in renal IRI was increased compared to the normal level, while the expression level of miR-25-3p was significantly downregulated and the changes in the other three microRNAs were statistically insignificant (Figure 2a). In addition, we verified this result via *in vitro* experiments. Compared with the control group, the expression level of miR-144 was upregulated in NRK-52E cells subjected to the H/R treatment, while the expression level of miR-25-3p was significantly downregulated (Figure 2b).

### 3.3 Role of miR-25-3p in NRK-52E cells during HRI

**Figure 3:**
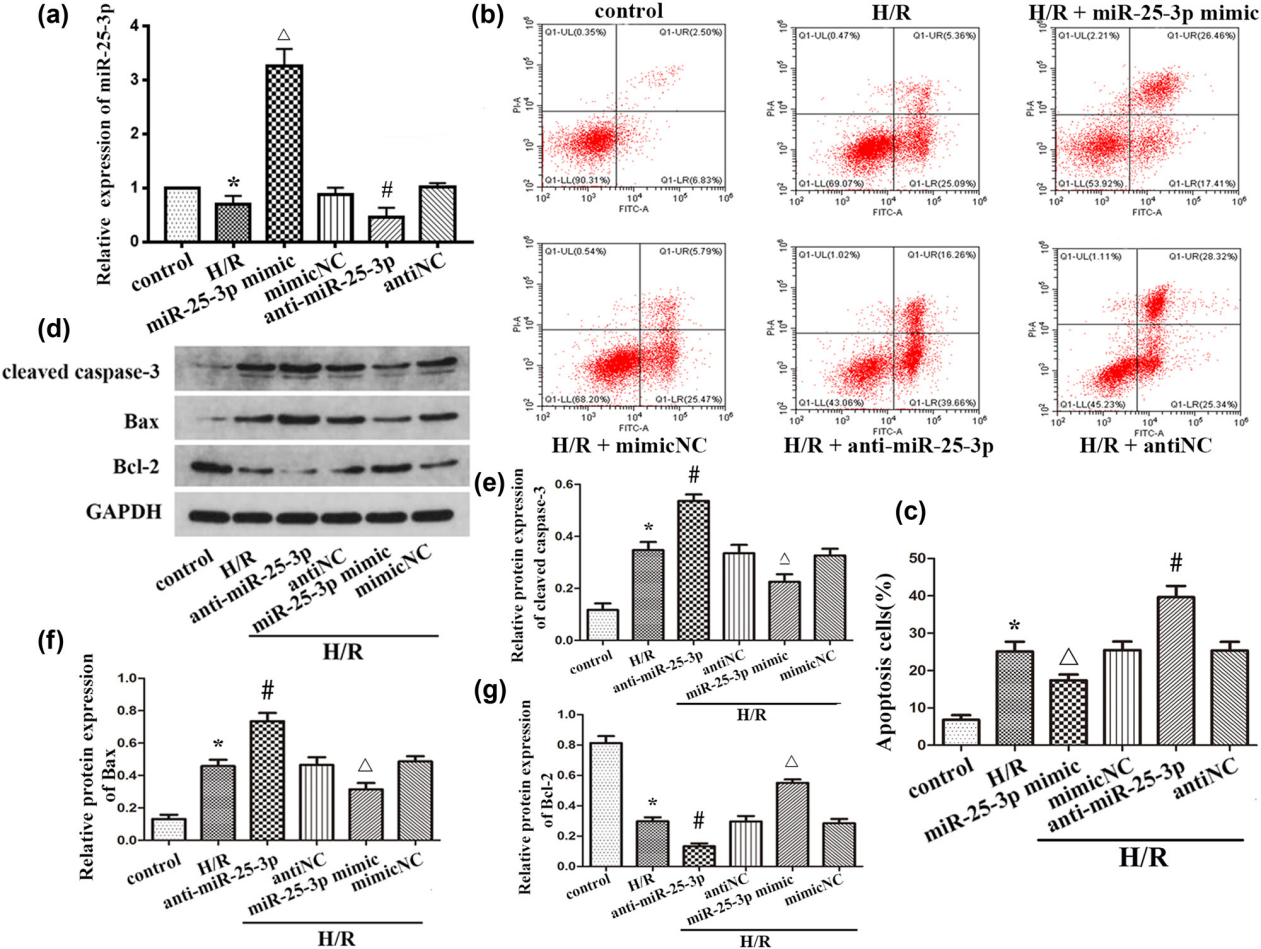
Overexpression of miR-25-3p can inhibit the expression of pro-apoptotic proteins and reduce hypoxia-induced apoptosis of renal tubular cells. (a) Expression of miR-25-3p in normal cells and NRK-52 cells subjected to H/R treatment after transfection with a mimic or inhibitor or corresponding controls. (b) Flow cytometry analysis shows the apoptosis of NRK-52E cells transfected with mimics or inhibitors of corresponding controls. (c) Quantitative analysis of the percentage of apoptosis of NRK-52E cells in each group. (d) Expression levels of apoptosis-related proteins (cleaved caspase-3, Bax, and Bcl-2). (e–g) Quantitative analysis of the relative expression levels of the cleaved caspase-3, Bax, and Bcl-2 proteins. The data are expressed as the mean ± SD. *P < 0.05 versus the control group, Δ*P* < 0.05 versus the H/R + mimicNC group, ^#^ *P* < 0.05 versus the H/R + antiNC group.

To further study the specific role of miR-25-3p in the progression of renal IRI, miR-25-3p mimics, inhibitors, or corresponding controls were transfected into NRK-52E cells under hypoxic conditions, and qRT-PCR was used to confirm the transfection efficiency (Figure 3a). The results showed that in cells transfected with the miR-25-3p mimic, miR-25-3p was significantly upregulated, while in cells transfected with the miR-25-3p inhibitor, miR-25-3p was significantly downregulated. In addition, subsequent flow cytometry analysis showed that H/R treatment significantly increased the apoptosis of NRK-52E cells while transfection of the mimic rescued this apoptosis. In contrast, transfection of miR-25-3p inhibitors aggravated hypoxia-induced apoptosis (Figure 3b and c). These results reveal that overexpression of miR-25-3p can protect renal tubular cells and prevent them from undergoing apoptosis during renal IRI. Western blot analysis was used to further explore the role of miR-25-3p at the protein level in renal IRI (Figure 3d). In the cells subjected to the H/R treatment, the apoptosis-related proteins cleaved caspase-3 and Bax were significantly upregulated while Bcl-2 was significantly downregulated. This situation was significantly increased after the inhibitor was transfected. In contrast, in the cells transfected with the mimic, the overexpression of miR-25-3p significantly reduced the expression levels of cleaved caspase-3 and Bax and enhanced the expression levels of Bcl-2 (Figure 3e–g). The above results indicate that miR-25-3p can reduce hypoxia-induced renal injury by inhibiting renal tubular cell apoptosis.

### 3.4 DKK3 is the direct target of miR-25-3p in NRK-52E cells during HRI

**Figure 4:**
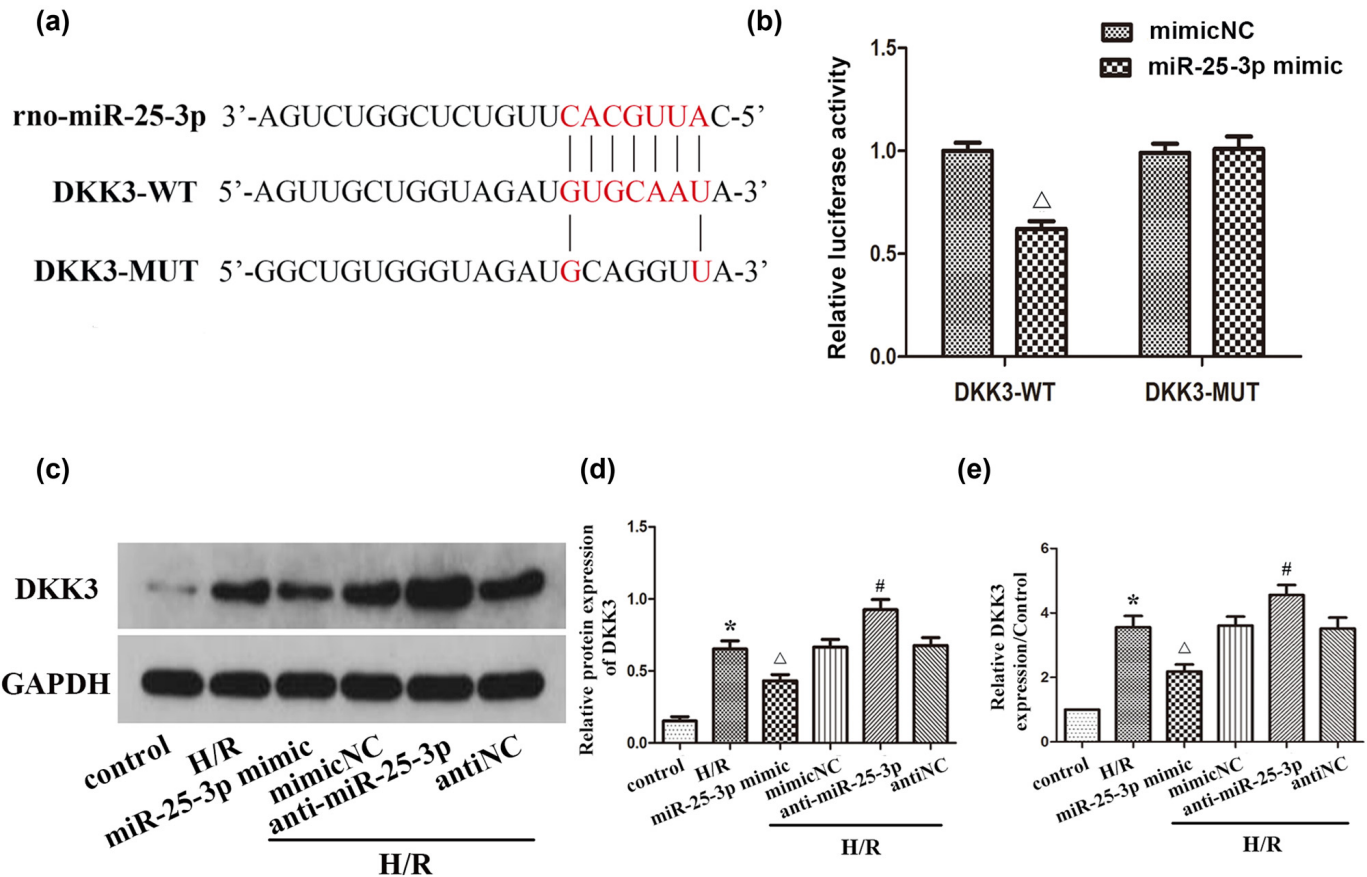
In NRK-52E cells, DKK3 is the direct target of miR-25-3p. (a) The TargetScan gene database predicts that there is a conservative binding site for miR-25-3p in the 3′-UTR of the DKK3 gene. (b) The luciferase reporter construct of wild-type DKK3 (DKK3-WT) or mutant DKK3 (DKK3-MUT) is co-transfected with miR-25-3p mimic or control into NRK-52E cells, and the luciferase activity is measured. (c) DKK3 protein expression level in cells transfected with miR-25-3p mimics or inhibitors. (d) Quantitative analysis of DKK3 protein expression level. (e) DKK3 mRNA expression level in cells transfected with miR-25-3p mimics or inhibitors. The data are expressed as the mean ± SD. *P < 0.05 versus the control group, Δ*P* < 0.05 versus the H/R + mimicNC group, ^#^ *P* < 0.05 versus the H/R + antiNC group.

To further explore the possible mechanism of miR-25-3p during HRI, a series of experiments were carried out to explore the pathways downstream of miR-25-3p. Through the online databases, TargetScan and miRDB, DKK3 was predicted to be a potential target gene of miR-25-3p. Bioinformatics methods were used to predict the binding site sequence of miR-25-3p and DKK3 and the mutation sequence of DKK3 (Figure 4a). To verify the relationship between miR-25-3p and DKK3, luciferase reporter gene constructs of mutant DKK3-MUT and wild-type DKK3 (DKK3-WT) carrying the DKK3 3 3′UTR were constructed. After transfecting luciferase reporter gene constructs into HRI-treated NRK-52E cells, the miR-25-3p mimic or mimicNC was transfected into the cells, and then the luciferase activity was measured. Compared with the mimicNC group, the luciferase activity of the miR-25-3p mimic transfection group was significantly decreased (Figure 4b). In addition, after hypoxic injury, the protein expression level of DKK3 in NRK-52E cells was significantly upregulated. After transfection of the mimic, the protein expression level of DKK3 decreased, and this change was reversed after the inhibitor was transfected (Figure 4c and d). The qRT-PCR results confirmed that the mRNA expression trend of DKK3 was consistent with the Western blotting results (Figure 4e). All these results indicate that DKK3 is a direct target of miR-25-3p.

### 3.5 Role of miR-25-3p in renal IRI

**Figure 5:**
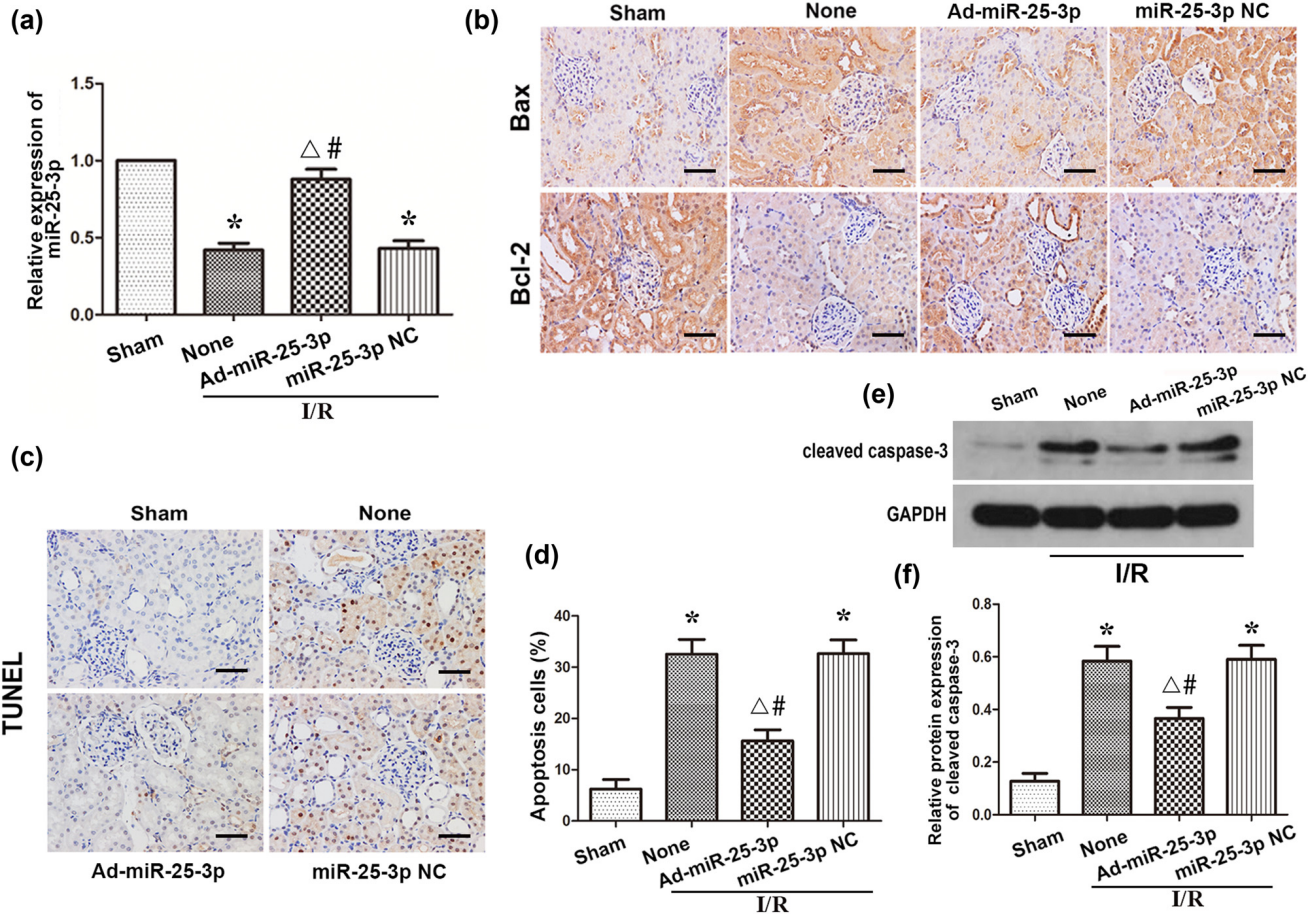
Overexpression of miR-25-3p in animals can reduce apoptosis during renal IRI. (a) The expression level of miR-25-3p in normal kidney and adenovirus-transfected kidney tissue. (b) Immunohistochemistry analysis of the Bax and Bcl-2 protein expression levels in kidney tissues from each group. The brown cells indicated by the black arrow are positively stained. Scale bar, 50 µm. (c) TUNEL staining of kidney tissues from each group. The brown-black cells indicated by the black arrows are TUNEL positive. Scale bar, 50 µm. (d) Quantitative analysis of the percentage of TUNEL-positive cells in each group. (e) Expression levels of cleaved caspase-3 in each group. (f) Quantitative values of the relative expression levels of cleaved caspase-3 proteins in each group. The data are expressed as the mean ± SD. * *P* < 0.05 versus the sham group, Δ*P* < 0.05 versus the I/R + None group, ^#^ *P* < 0.05 versus the I/R + miR-25-3p NC group.

To study the effect of miR-25-3p on hypoxia-induced AKI, we administered ad-miR-25-3p and ad-miR-Con to Sprague-Dawley rats via tail vein injection, and all rats transfected with adenovirus were treated with renal ischemia and reperfusion 3 days later. The kidney tissues of each group were collected for subsequent experiments. qRT-PCR was used to detect the expression level of miR-25-3p in the tissues. The results showed that the expression level of miR-25-3p in the I/R + ad-miR-25-3p group was significantly upregulated compared to the I/R + None group. However, this difference did not exist between the I/R + ad-miR-25-3p group and the I/R + None group (Figure 5a). These results indicated that the *in vivo* transfection experiment was successful. Compared with the sham group, the percentage of Bax-positive cells was significantly increased in the I/R + None group, while the percentage of cells positively stained for Bcl-2 was downregulated; however, this change was reversed after overexpression of miR-25-3p (Figure 5b). The kidney tissues that had undergone TUNEL staining were observed under a light microscope, and the number of TUNEL-positive cells was counted. The results showed that the number of apoptotic cells in the I/R + None group was significantly increased compared with that in the sham group (Figures 5c and d). Consistent with these results, the Western blot analysis showed that the protein expression levels of cleaved caspase-3 in the I/R + None group were significantly higher than those in the sham group; however, this situation was reversed in the I/R + ad-miR-25-3p group (Figures 5e and f). In summary, the above results all indicate that overexpression of miR-25-3p in animals can reduce the expression level of proapoptotic proteins in the kidney and reduce cell apoptosis during renal IRI.

## 4 Discussion

Renal IRI is common after kidney transplantation, and it severely affects the prognosis of the disease by inducing the loss of renal tubular cell function. Studies have shown that the accumulation of oxygen free radicals and the apoptosis of renal tubular cells caused by inflammation are the main causes of renal IRI [19–21]. In recent years, an increasing number of studies have shown that the Wnt/β-catenin pathway plays an important role in renal cell protection after renal IRI. As a negative regulator of the Wnt/β-catenin signaling pathway, DKK3 plays an important regulatory role in many kidney diseases [22–24].

Dickkopf (Dkk) genes are an evolutionarily conserved small gene family consisting of four members (Dkk1-4) and a unique Dkk3-related gene (Dkkl1). The secreted proteins encoded by the Dkk family are glycoproteins and mainly composed of Dkk1-4 [25]. A large number of studies have confirmed that the main role of Dkk genes is to target the Wnt signaling pathway, especially the Wnt/β-catenin pathway, which has long been proven to play an important role in kidney disease [26–28]. Wnt molecules bind to frizzled receptors and coreceptor LDL receptor-related protein (Lrp) 5/6 to transmit signals within the cell. This combination can inhibit the phosphorylation of β-catenin by the destruction complex and the subsequent ubiquitination and degradation of phosphorylated β-catenin and promote the accumulation of nonphosphorylated β-catenin in the cytoplasm. Then, β-catenin is transferred to the nucleus and engages DNA-bound TCF transcription factors to promote the transcription of Wnt target genes. In the process of renal IRI, the Wnt/β-catenin pathway is activated and plays a protective role. It promotes the expression of downstream proteins such as c-myc and cyclin D1 to reduce cell damage and promote cell survival. Studies have confirmed that Wnt agonists improve renal regeneration and kidney function after renal IRI while also reducing inflammation and oxidative stress in the kidney [29]. However, in renal IRI, Dkk3 inhibits Wnt signal transduction by competitively binding with Lrp5/6 and inhibits the protective effect of the Wnt/β-catenin pathway on the kidneys in AKI. After the Wnt/β-catenin pathway is inhibited, the decrease in the level of β-catenin in the cytoplasm can directly induce the increase in renal expression of p53 and Bax and the decrease in Akt phosphorylation and survivin expression, which can lead to renal cell apoptosis and functional impairment. In addition, more studies have shown that the levels of macrophages and various pro-inflammatory cytokines in DKK 3 −/− ApoE −/− mice are significantly lower than those in ApoE −/− mice. This reveals that the knockout of DKK3 can significantly reduce inflammation and reduce cell damage [30]. Research by Li et al. confirmed that overexpression of DKK3 can inhibit cell proliferation, induce cell apoptosis and inhibit collagen synthesis through the TGF-β1/Smad signal axis [31]. Although a large number of studies have revealed that DKK3 plays an important role in a variety of disease processes, and this role is inseparable from the regulation of cell apoptosis, inflammation, and proliferation, studies on the role and mechanism of DKK3 in renal IRI are still few.

miRNAs are important regulators in the body. A large number of studies have shown that they play an important role in many diseases by targeting target genes to regulate cell apoptosis, proliferation, oxidative stress, and the release of inflammatory factors [32–35]. In recent years, an increasing number of studies have shown that miR-25-3p plays an important regulatory role in IRI. Liu et al. confirmed that miR-25 can inhibit cell apoptosis and the release of inflammatory factors by targeting HMGB1 and ultimately play a protective role in myocardial IRI [17]. However, there are few studies on the role and mechanism of miR-25-3p in renal IRI. In this study, by co-transfecting luciferase reporter gene constructs carrying the DKK3 3′UTR with the miR-25-3p mimic, we confirmed that DKK3 is the direct target of miR-25-3p. In *in vitro* experiments, the expression level of DKK3 in miR-25-3p mimic-transfected cells was significantly downregulated compared with that in the anti-miR-25-3p group. Consistent with the aforementioned results, the same results were obtained in *in vivo* experiments. All these results indicate that miR-25-3p has a negative regulatory effect on DKK3. Research by Li et al. showed that miR-25-3p can inhibit oxidative stress to play the role of anti-epileptic [36]. In addition, miR-25 has also been confirmed to reduce the activity of nuclear factor-kappaB and the transcriptional activation of TNF-α and IL-6 and inhibit the migration of macrophages [37]. However, this study focused on exploring the regulatory effects of miR-25-3p on cell apoptosis, and its regulatory effects on oxidative stress and inflammation in renal IRI still need to be further explored.

In this study, we first simulated hypoxia and reoxygenation models with different reoxygenation times at the animal and cell levels and selected 24 h of reoxygenation for animals and 4 h of cell reoxygenation for subsequent experiments. Then, we tested the expression level of miR-25-3p during renal IRI. The results showed that miR-25-3p was significantly downregulated in kidney tissues receiving IRI and NRK-52E cells undergoing HRI. Further analysis showed that overexpression of miR-25-3p can significantly reduce the expression of proapoptotic proteins and inhibit hypoxia-induced apoptosis of NRK-52E cells. In contrast, inhibiting the expression of miR-25-3p exacerbates hypoxia-induced apoptosis. Zhang et al. showed that miR-25-3p, as a negative regulator of the Fas/FasL pathway, increased its expression level to inhibit cerebral IRI-induced neuronal apoptosis [38]. The evidence we provide is consistent with Zhang’s discovery that overexpression of miR-25-3p can effectively reduce hypoxia-induced apoptosis. Subsequently, we used adenovirus to regulate the expression level of miR-25-3p in rats. Through many detection methods applied here, we obtained results consistent with *in vitro* experiments.

However, related experiments are still needed, such as silencing DKK3 at the cellular level to explore whether DKK3 is a key target gene for miR-25-3p to achieve protection, and experiments using DKK3 gene knockout rats are urgently needed. In conclusion, it is reasonable to believe that miR-25-3p protects kidney cells from apoptosis in renal IRI by targeting DKK3.
